# Supplementary figures and images for: The pathogenesis of experimental Emergomycosis in mice
Source: PLoS Negl Trop Dis. 2024 Jan 10;18(1):e0011850. doi: 10.1371/journal.pntd.0011850 (PMC10805315; doi:10.1371/journal.pntd.0011850)

S1 Fig

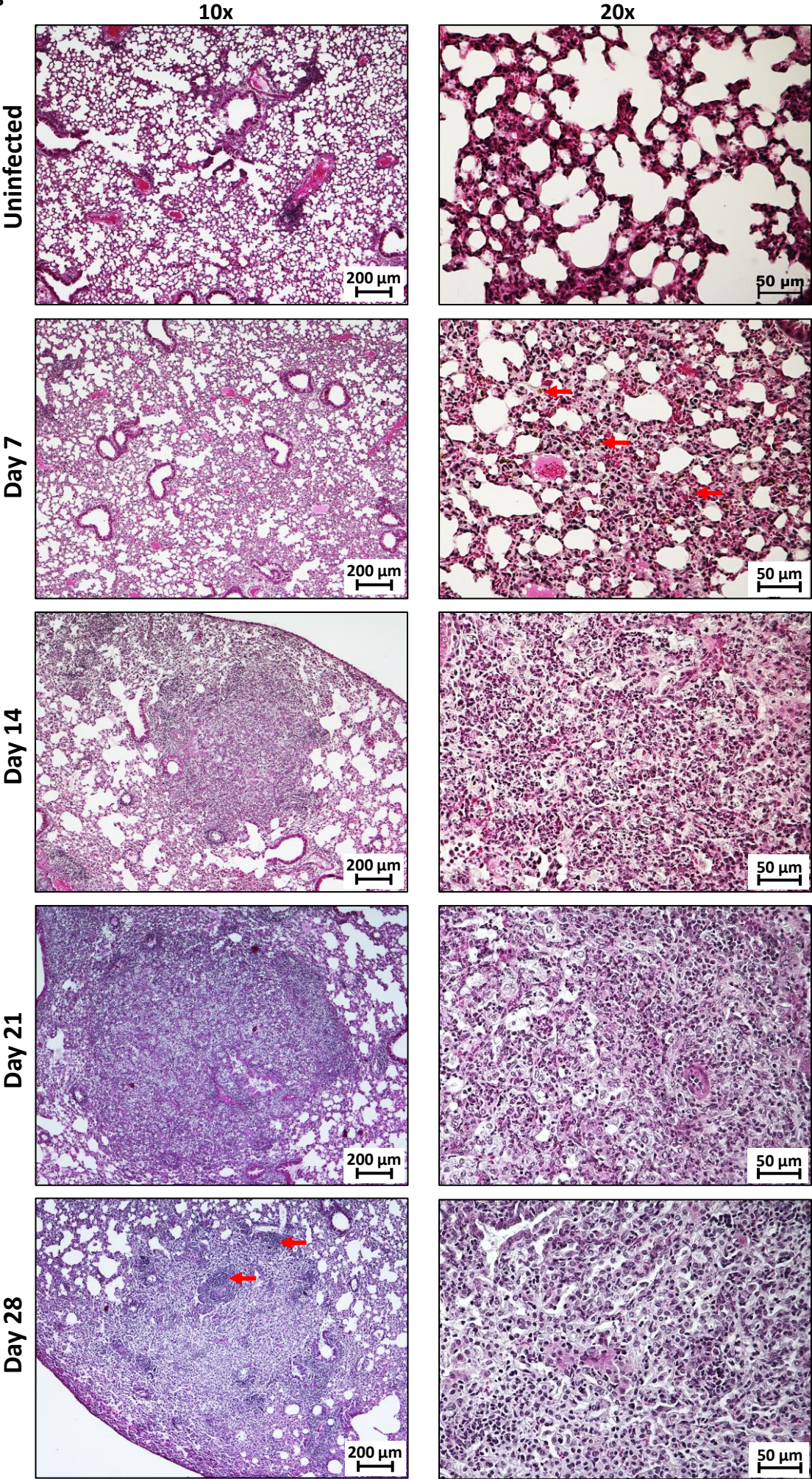

Supplement: S1 Fig — C57BL/6 mice were infected with 1x10^3^ yeast cells in 50 μl PBS and euthanized at weekly intervals post-infection. Sections were stained with H&E for inflammation and represented photomicrographs are shown for 10x and 20x magnification. (B) Representative images of lung sections stained with GMS at 10x and 20x magnification. (PDF) [file pntd.0011850.s001.pdf]

S2 Fig

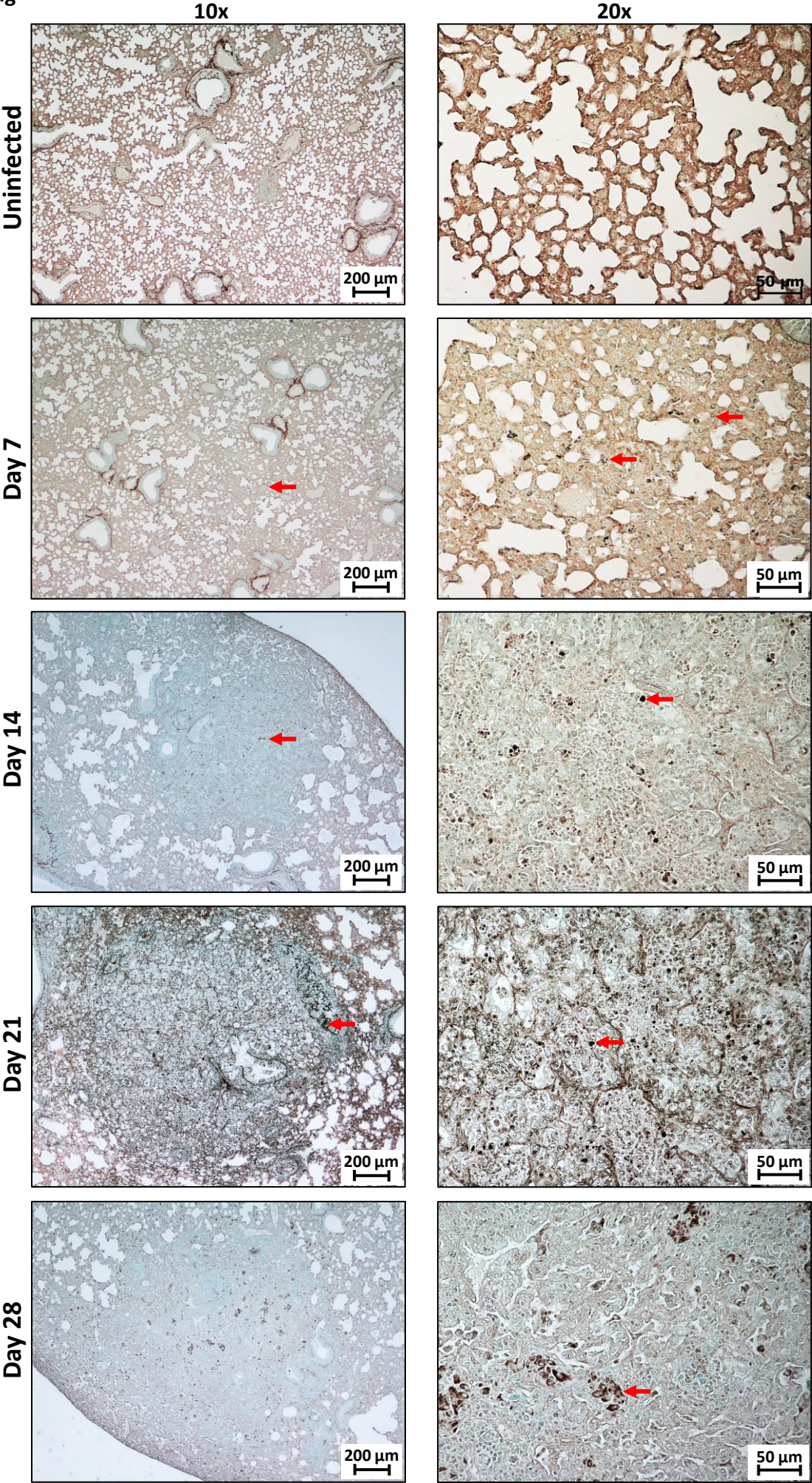

Supplement: S2 Fig — C57BL/6 mice were infected with 1x10^3^ yeast cells in 50 μl PBS and euthanized at weekly intervals post-infection. Sections were stained with GMS for fungal organisms and representative photomicrographs are shown for 10x and 20x magnification. GMS: Grocott methenamine silver. (PDF) [file pntd.0011850.s002.pdf]

S3 Fig

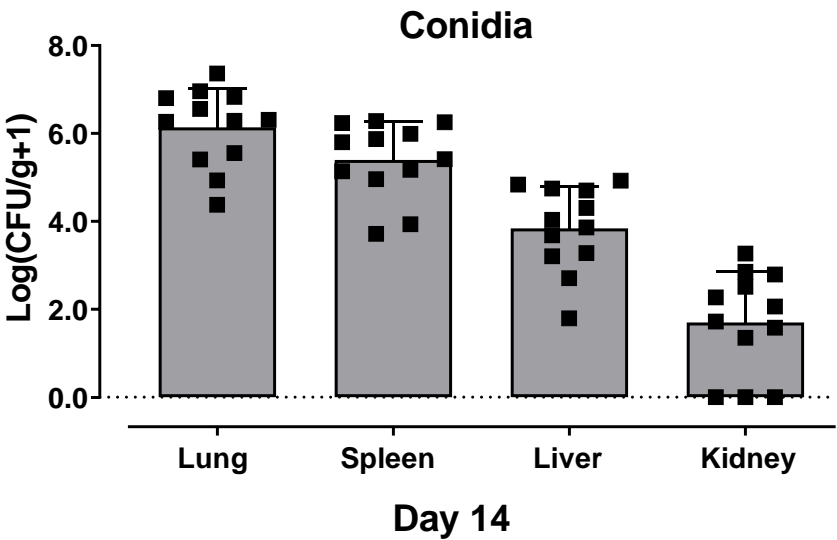

Supplement: S3 Fig — C57BL/6 mice were infected with 1x10^3^ conidia cells in 50 μl PBS and euthanized at Day 14 post-infection. Conidia infection resulted in the dissemination of organisms from the lung to the spleen, liver, and kidney shown by CFU per gram tissue. Data represents two independent pooled experiments, n = 6 mice per experiment. (PDF) [file pntd.0011850.s003.pdf]

Lung Day 14 post infection

C57BL/6

Rag-1<sup>-/-</sup>

H&E

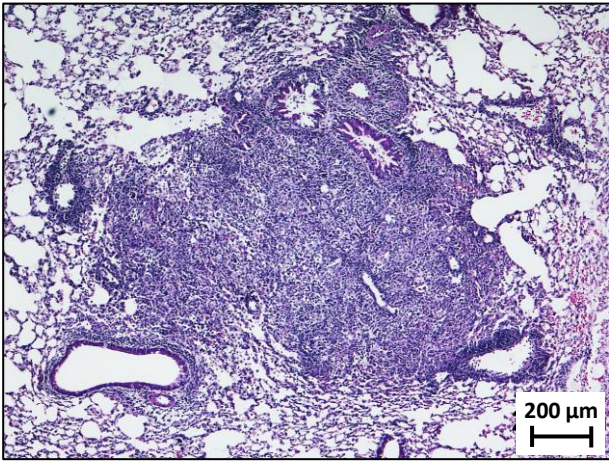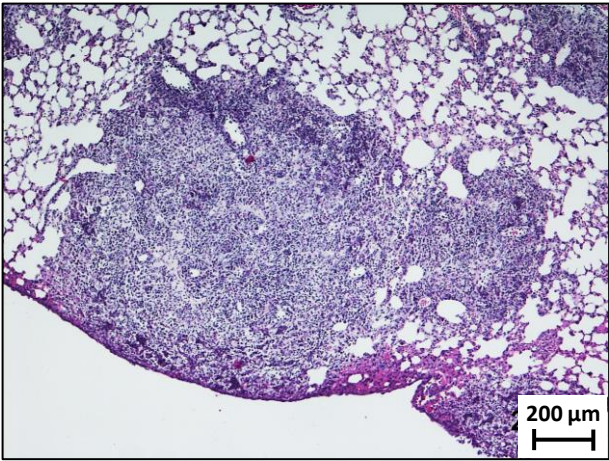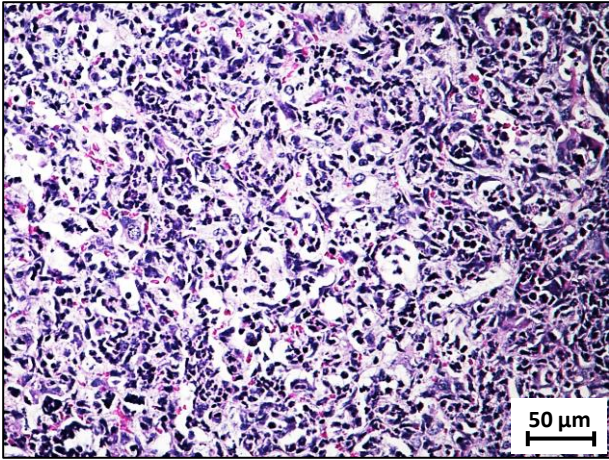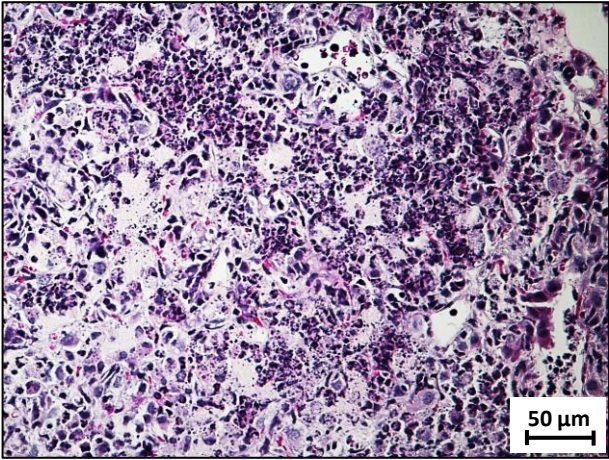

GMS

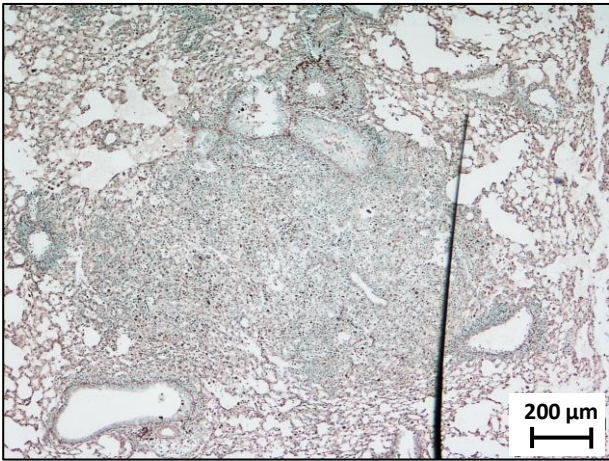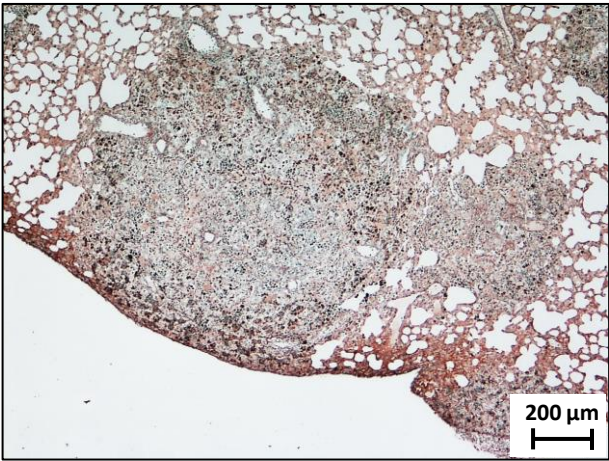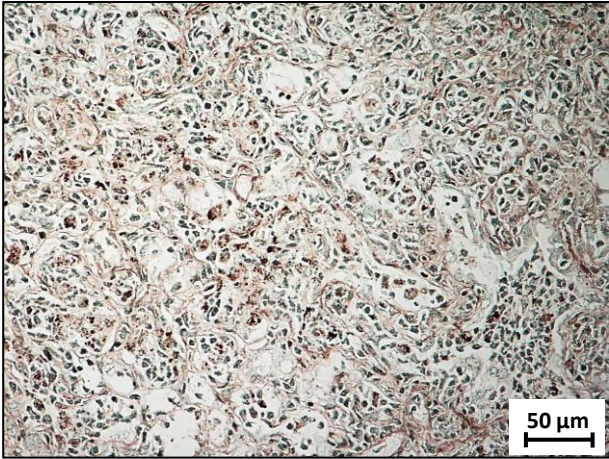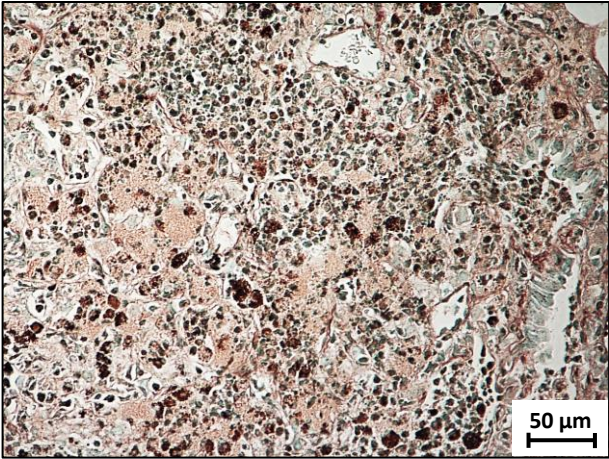

Supplement: S4 Fig — C57BL/6 and Rag-1-/- mice were infected with 1x10^3^ yeast cells and euthanized at Day 14 post infection. Representative images of lung sections from C57BL/6 and Rag-1-/- mice stained with H&E and GMS at 10x and 20x magnification. H&E: hematoxylin and eosin; GMS: Grocott methenamine silver. (PDF) [file pntd.0011850.s004.pdf]

S5 Fig

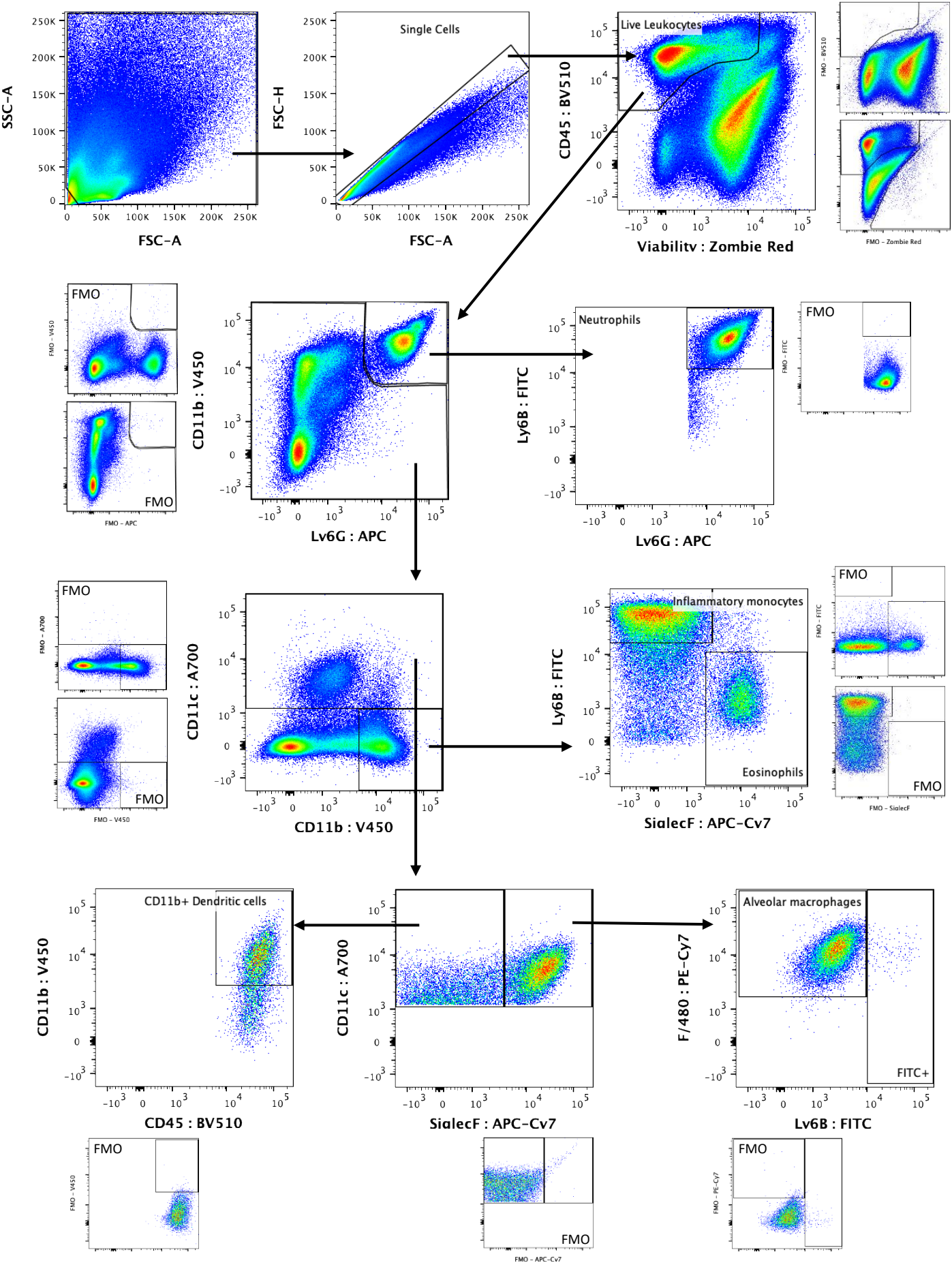

Supplement: S5 Fig — C57BL/6 and Rag-1-/- mice were infected with 1x10^3^ yeast cells. Lungs were harvested at Day 14 post-infection. The myeloid gating strategy for infected lung tissue was initiated by excluding cell debris and gating for single cells. Live leukocytes were identified as CD45+ and Zombie Red-. Myeloid cell populations were gated as follows: neutrophils (CD11b+, LY6G+, LY6B+), inflammatory monocytes (CD11b+, LY6B+, SiglecF-neg), eosinophils (CD11b+, SiglecF+, LY6B-neg), alveolar macrophages (CD11c+, SiglecF+, F4/80+) and CD11b+ dendritic cells (LY6G-neg, CD11c+, CD11b+) (B) (see below). (PDF) [file pntd.0011850.s005.pdf]

S6 Fig

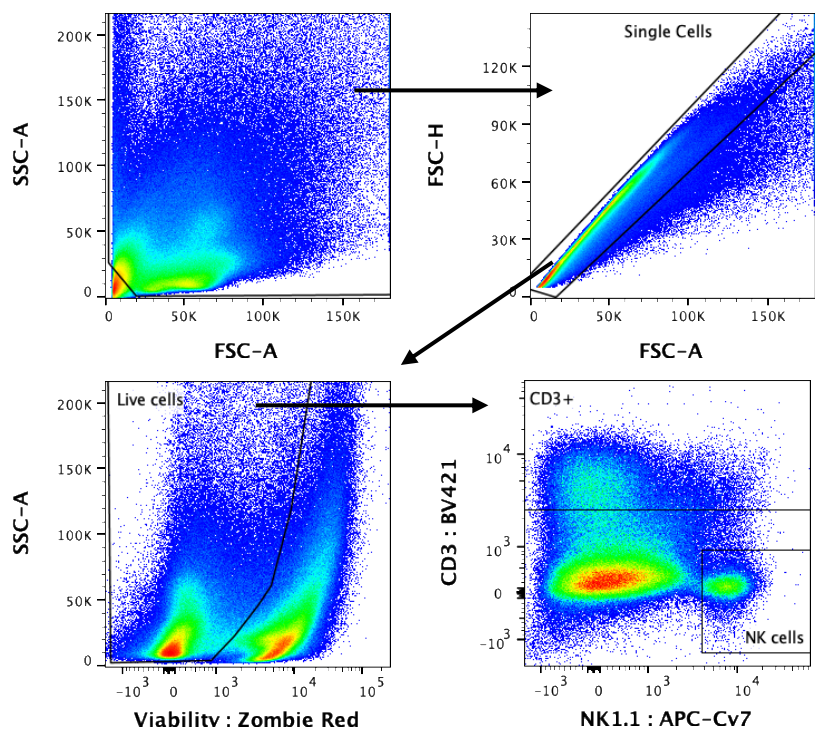

Supplement: S6 Fig — C57BL/6 and Rag-1-/- mice were infected with 1x10^3^ yeast cells. Lungs were harvested at Day 14 post-infection. Separate staining was done to identify NK cell populations in both mouse strains; gating for NK cells: (CD3-neg, NK1.1+). FMO: fluorescence minus one; NK: natural killer. (PDF) [file pntd.0011850.s006.pdf]

S7 Fig

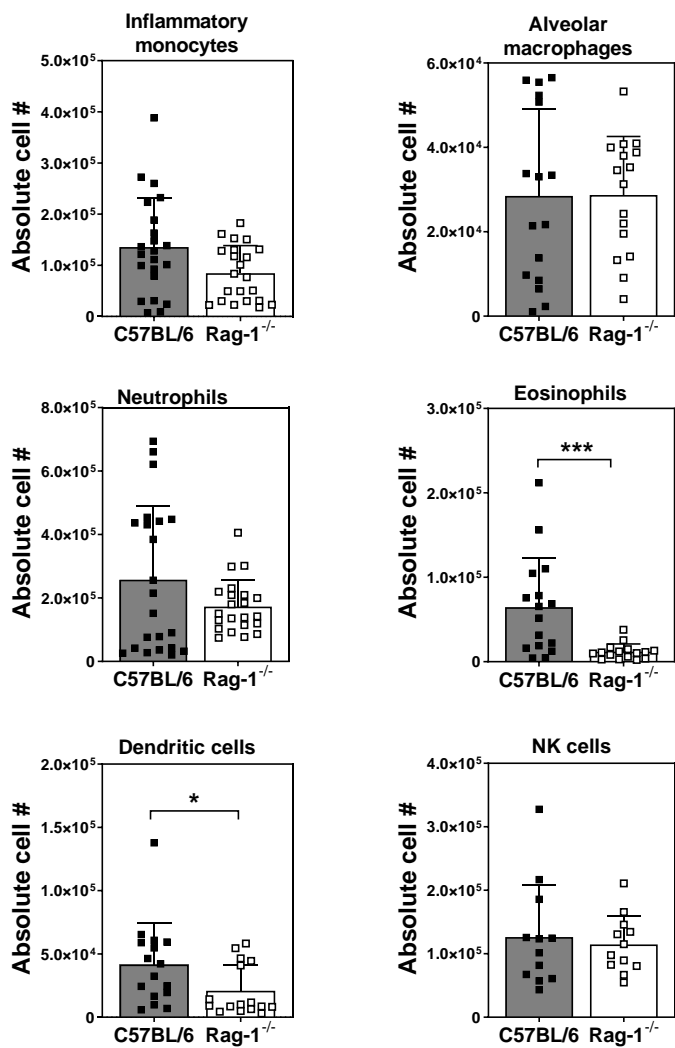

Supplement: S7 Fig — C57BL/6 and Rag-1-/- mice were infected with 1x10^3^ yeast cells and lungs harvested at Day 14 post-infection. Absolute cell numbers shown for myeloid cell populations identified as follows: neutrophils (CD11b+, LY6G+, LY6B+), inflammatory monocytes (CD11b+, LY6B+, SiglecF-neg), eosinophils (CD11b+, SiglecF+, LY6B-neg), alveolar macrophages (CD11c+, Siglecf +, F4/80+) and CD11b+ dendritic cells (LY6G-neg, CD11c+, CD11b+). Separate staining for NK cell populations in both mouse strains; gating for NK cells: (CD3-neg, NK1.1+). Data represents three-four pooled experiments, n = 4–5 mice/group per experiment, mean ± SD, *p<0.05 and ***p<0.001 C57BL/6 vs. Rag-1-/- (student t-test). FMO: fluorescence minus one, NK: natural killer. (PDF) [file pntd.0011850.s007.pdf]
