# Supplementary material for: The pathogenesis of experimental Emergomycosis in mice
Source: PLoS Negl Trop Dis. 2024 Jan 10;18(1):e0011850. doi: 10.1371/journal.pntd.0011850 (PMC10805315; doi:10.1371/journal.pntd.0011850)
Supplement: S1 Table — This table provides a list of FACS antibodies used to identify cell types. (PDF) [file pntd.0011850.s008.pdf]

S1 Table: Flow cytometry antibody information

Target Protein	Species	Conjugation	Clone	Company
Ly6G	rat anti-mouse	APC	1A8	BD Pharmingen
CD11c	hamster anti-mouse	Alexa Fluor® 700	HL3	BD Pharmingen
CD11b	rat anti-mouse	V450	M1/70	BD Horizon
neutrophil (7/4)	rat anti-mouse	FITC	GR288279-11	Abcam
CD45	rat anti-mouse	BV510	30-F11	BD Horizon
F4/80	rat anti-mouse	PE/Cy7	BM8	Biolegend

*Zombie Red™ Fixable Viability Kit (Biolegend, USA). **Blocking agents were added to antibody cocktails to prevent non-specific binding of the antibodies: eBioscience™ normal rat serum (Invitrogen, USA) and mouse BD Fc block™ rat anti-mouse CD16/CD32 antibody (clone:2.4G2, BD Pharmingen).
