# Supplementary material for: The pathogenesis of experimental Emergomycosis in mice
Source: PLoS Negl Trop Dis. 2024 Jan 10;18(1):e0011850. doi: 10.1371/journal.pntd.0011850 (PMC10805315; doi:10.1371/journal.pntd.0011850)
Supplement: Attachment — Submitted filename: cover letter and response.pdf [file pntd.0011850.s009.pdf]

Associate Professor J. Claire Hoving

Wellcome Trust Intermediate Fellow Public Health and Tropical Medicine
Fungal Immunology Group, AFGrica
Institute of Infectious Disease and Molecular Medicine
Room 3.06.1 Werner & Beit South Building,
Faculty of Health Sciences
Mobile: +27(0)82 304 8246
E-mail: jennifer.hoving@uct.ac.za
Internet: www.idm.uct.ac.za

24 October 2023

Dear Professor's Kamhawi, Brindley and Hotez,

Re: Revision of manuscript PNTD-D-23-01083

Thank you for considering our manuscript titled, "The pathogenesis of experimental emergomycosis in mice", for submission to the journal *PLoS Neglected Tropical Diseases*.

We are delighted that the reviewers feel our work warrants publication in your journal and appreciate the constructive feedback provided by the reviewers. We hope to have fully addressed their concerns in our point-to-point response below and have made the necessary revisions to the manuscript. The most prominent revision was the "Discussion" where we reduced the comparison with *Histoplasma capsulatum* infection to provide a clear distinction between our own work and that published on other thermally dimorphic fungal pathogens.

We look forward to hearing your response and hope that this work will be considered for publication.

Sincerely,

Assoc. Professor J. Claire Hoving

Point-to-point response to reviewer's coments:

Reviewer #1: IL 10 results can be presented in the abstract as key cytokine when addressing IFN-gamma and immunopathogenesis.

Response:

Thank you for this comment, we have included the following sentence in the abstract:

Line 36: "No significant differences in TNF, IL-12p70 and IL-10 were observed in wild-type mice between one and four-weeks post-infection."

Reviewer #2: There is a spelling error on line 310.

Response:

"clinically-isolated"

Changed to:

"clinically isolated"

Reviewer #2: However, there were weaknesses in the study: the association with advanced HIV in the experimental model was not shown, therefore, I think that associations with HIV are only speculative as the authors did not infect the mice with HIV. There should be a consideration of the weaknesses of rodent models for HIV pathogenesis and how this impacts the presentation of other pathologies associated with HIV infection.

Response:

We have included this limitation into our discussion as follows:

Line 458:

"While we can use these observations to understand the host response during infection and how a weakened immune response may promote disease, we can only speculate the outcome during advanced HIV disease. Perhaps this could provide an explanation as to why we did not observe cutaneous lesions in mice as seen in human infection. This may be specific to advanced HIV patients with emergomycosis. Future work could include humanized mice infected with HIV or establishing a dermal infection model, infecting mice by skin abrasions rather than a pulmonary infection route. Despite these limitations, the animal model of E. africanus is an indispensable step forward in understanding the pathogenesis and immune components required for clearing infection."

Reviewer #2: Why did the authors only focus on immunoanalyses of innate immune cells? Especially given that they proceed to use Rag-1 deficient mice which have defective adaptive immune systems?

Response:

As emergomycosis is only found in patients with a low CD4 count, and therefore immunocompromised, for this first manuscript it was important to define the model in an immunocompromised host which lacks CD4+ T cells. Our future work would include a kinetic study of the recruited cells in wildtype mice at both early and late timepoints to fully characterise the innate and adaptive responses. Here we will also compare the immune response to both yeast cells and conidia and determine how early conidia can switch to yeast form and how this affects the immune response. In addition, intracellular cytokine staining would provide insights into adaptive cell-specific cytokine production and functionality.

Reviewer #2: What would be the importance of the significantly lower eosinophils in the Rag 1-/- mice that had worse disease?

Response:

We showed significantly lower eosinophils in the lung of Rag-1-/- mice. Localized lung inflammation and inflammatory cytokine and chemokine production from macrophages, also recruits other innate cells such as neutrophils, dendritic cells, innate lymphoid NK cells and eosinophils. However, we speculate that decreased eosinophils may not be contributing to worse disease outcome. We suggest that it is rather the lack of adaptive immune responses and effector immune functions leading to uncontrolled infection and defective clearance.

Reviewer #2: Also, it is noted that the cytokines are produced a week after infections despite a large fungal burden. How is this mediated and why is the response so delayed? Is there another player of the adaptive immune system that may be triggering these responses in the lung?

Response:

Indeed, it is interesting to note that there are minimal inflammatory cytokine responses in the lung at one week post infection, despite a large fungal burden, suggesting that *Es. africanus* may elicit a minimal innate immune response unlike other opportunistic fungal pathogens. Innate immune cells are known to produce pro-inflammatory cytokines, increasing cellular recruitment to the site of infection. Dendritic cells travel to draining lymph nodes for antigen presentation to T cells. Our data shows a significant increase in IL-12p40 at two weeks post-infection. IL-12 production is known to stimulate T cell activation and differentiation into Th1 cells which also infiltrate the site of infection and form an important component of the granuloma structures. During adaptive immunity, Th1 cells release IFN-y (which we show peaks at three weeks post-infection) in response to infection which importantly activates innate immune cells such as macrophages to induce effector functions which aid in the killing of internalised yeast cells. This peak in IFN-y production in the homogenised lung supernatant at three weeks post-infection, is likely in line with the activation of the adaptive immune responses between two to three weeks. However further investigation of adaptive immune responses and intracellular cytokine staining by flow cytometry in needed to gain additional insights.

Reviewer #2: How closely does the widespread infection in mice (in multiple tissues) compare to the human case? Was this a hyperbolic response? If so, how can one interpret results from these models in a human context? Was there a reason humanized mice (with a reconstitution of the human immune systems) were not used in these studies?

Response:

There are limitations to our model, mentioned in the discussion confounded by the very limited data about the mechanism of disease in humans. And while we have not included HIV-infection, we could extrapolate our observations, particularly with the reduction in IFN-y, to humans in a reverse translational approach. For example, our next step would be to determine if there is a systemic reduction in IFN-y in the mice (blood or spleen) and then measure IFN-y in patients with advanced HIV disease (with or without emergomycosis) to determine if there is a correlation between IFN-y levels and enhanced susceptibility to infection. Humanised mice are versatile tools for the study of HIV and its interaction with the human immune system. Combining this model with our emergomycosis model would provide key insights into the role co-infection plays in human responses and pathogenesis. However, our work describes a murine model of *Emergomyces* spp. infection for the first time, and initially it was not clear if *Emergomyces* spp. could even infect mice. Therefore, this model provides the foundation for future work and provides insights into further avenues to explore mechanisms of pathogenesis, including a humanised mouse model of HIV.

Reviewer #2: What about the immune response in other tissues? Of interest is the persistent infection in the spleen, an immunoregulatory site... why was this evident? Some speculation on this would be of interest.

Response:

Clearance of CFU in the spleen was likely just delayed and not a persistent infection as no detectable CFU was found in mice that survived long term. The mice that did not succumb to infection during survival experiments were euthanized at week 18 (n=7) and week 46 (n=11) and no detectable CFU were found in the lung or spleen. We show effective granuloma formation in the lungs of both immunocompetent and immunocompromised mice, which we know is advantageous in limiting widespread tissue infection and localising effector immune responses. We do not however see granuloma formation in the splenic tissue, but rather see diffuse yeasts throughout the tissue, possibly leading to the increased fungal burden and delayed clearance we see, however, this would require further investigation.

Reviewer #2: A lot of comparisons are made with H. capsulatum infections in the discussion. These comparisons confuse the reader and sometimes I am not sure if the paper was to establish a murine model of E. africanus infections or to compare and contrast its pathology to known H. capsulatum infections. Why would we care about H. capsulatum infections anyway in the context of advanced HIV?

Response:

We thank the reviewer for this observation and significantly reduced the comparison with *Histoplasma* to provide a clear distinction between our own work and that published on other dimorphic pathogens as follows:

Removed lines 313-316: ~~"Since little is known or published about the host immune response to *Es. africanus*, we compared our results and observations with other closely related thermally-dimorphic fungi, specifically, *H. capsulatum* due to its phylogenetic and genetic similarity to *Emergomyces* spp"~~

Removed lines 340-344 and associated reference: ~~A similar study comparing disease kinetics of multiple H. capsulatum strains in C57BL/6 mice infected with a similar dose (2.5x103 yeast cells per mouse) demonstrated a comparable weight loss profile, with lung CFU peaking 8- days post-infection for all H. capsulatum strains. Clearance of fungal organisms in the lung followed a similar trend with CFU below the detection limit by 32-days post-infection (Sepúlveda, Williams et al. 2014).~~

Lines 347-351: "Based on observations in other thermally dimorphic pathogens ~~W~~we speculate that granuloma formation during emergomycosis is induced by the infection of macrophages by yeast cells within the lung tissue, causing an infiltration of immune cells~~, which has also been demonstrated during histoplasmosis~~ (Heninger, Hogan et al. 2006)."

Lines 357-360 and reference: ~~While further investigation is required to confirm these observations, suppressing apoptosis during Histoplasma spp. infection has been shown to increase the severity of disease, associated with increased IL-10 and IL-4 production (Allen 2005).~~

Line 378: ~~(including *H. capsulatum*)~~

Line 380-382 and reference: ~~In *Histoplasma* spp., infection, neutralisation of IL-12 led to fatality and was associated with a decrease in IFN-γ, but not TNF or GM-CSF (Zhou, Sieve et al. 1995). Similar future studies in our E. africanus model, will determine if IL-12 plays a protective role.~~

Line 394-402: "Our data demonstrated an increase in IL-17 production at two-weeks post-infection. However, there is conflicting data on the role of IL-17 in fungal clearance. On the one hand, ~~Lastly,~~ increased IL-17 production by CD4+ T cells and activated macrophages has~~ve~~ been shown to induce macrophage effector function during *H. capsulatum* infection (Heninger, Hogan et al. 2006). In contrast~~, However, experiments in~~ C-type lectin receptor-deficient mice ~~showed a~~with a decrease in lung-specific IL-17~~but the~~were still able ~~mice maintained their ability~~ to clear infection. Therefore, we hypothesize that although IL-17 was increased ~~at two-weeks post-infection~~, it may not be essential and rather that Th1 responses are central in driving clearance.

Line 410-412 and references: ~~Although, B cell-deficient mice infected with H. capsulatum were not more susceptible to disease (Chandler, Smith et al. 1969, Allendörfer, Brunner et al. 1999).~~

Line 424: "….cell type in ~~*H. capsulatum*~~ fungal-mediated granuloma formation"

Line 426-445: "IFN-γ has been shown to play a major role in ~~all~~ dimorphic fungal immunity, with IFN-γ being crucial for the activation of macrophages, which play a central role in controlling the infection. ~~. Indeed,~~ For example, IFN-γ-deficient mice were shown to be highly susceptible to succumb to *H. capsulatum* infection ~~with significantly higher lung CFU, associated with significantly higher TNF and GM-CSF but not IL-12 or IL-4 cytokines~~ (Allendoerfer and Deepe 1997, Clemons, Darbonne et al. 2000). The source of ~~Multiple studies show that CD4+ and CD8+ T cells play a vital role in H. capsulatum pulmonary and systemic infection. Particularly, it is the production of~~ IFN-γ production is likely from ~~by~~ CD4+ T cells that drive macrophage activation and fungal clearance. Our work demonstrates that infected Rag1-/-Amice were able to produce ~~lthough~~ granuloma-like formations ~~were observed~~ in the lungs. However, of Rag-1-/- mice, these were likely predominantly composed of macrophages. ~~However~~ due to the lack of infiltrating CD4+ and CD8+ T cells, macrophages we~~a~~re probably unable to effectively kill intracellular

yeasts which leads to enhanced~~the enhanced and uncontrolled~~ disseminated infection and ~~eventual~~ death. in Rag-1-/- mice. Although significant, an even greater decrease in Rag-1-/- IFN-γ was expected. While IFN-γ production was significantly decreased in Rag-1-/- mice, considering the enhanced susceptibility, one would anticipate a more exaggerate decrease in this cytokine. An explanation is that ~~This observation may be explained by~~ the ~~presence and~~ increase in~~of~~ lymphoid NK cells ~~in Rag-1-/- mice which can also~~ produce IFN-γ. However, this is ~~ese levels may not be~~ insufficient to arm all macrophages and inhibit the intracellular growth of yeasts."

Reviewer #2: Most patients with advanced HIV are also on ARVs. What would be the impact of ARV treatment on E. africanus infections?

Response:

E. africanus infection is limited to severely immunocompromised patients (with CD4+ T cell count < 200 cells/mm^3). Therefore, patients receiving effective antiretroviral therapy (ART) are not likely to be susceptible to *Emergomyces* spp. as they would have controlled HIV viral loads and restored immune function. Of concern are the patients with ART treatment failure. An estimated 30% of HIV+ patients do not experience normalization of CD4+ T cell counts, particularly if ART was not initiated soon after HIV infection, leading to immune damage, T cell exhaustion and impartial immune reconstitution. There are no studies currently investigating the effect ART has on *Emergomyces africanus* itself. In fact, the optimal antifungal treatment strategy has not been established.
